# Vesicle-Mediated Control of Cell Function: The Role of Extracellular Matrix and Microenvironment

**DOI:** 10.3389/fphys.2018.00651

**Published:** 2018-06-05

**Authors:** Gorjana Rackov, Noemi Garcia-Romero, Susana Esteban-Rubio, Josefa Carrión-Navarro, Cristobal Belda-Iniesta, Angel Ayuso-Sacido

**Affiliations:** ^1^IMDEA Nanoscience Institute, Madrid, Spain; ^2^Fundación de Investigación HM Hospitales, Madrid, Spain; ^3^Facultad de Medicina (IMMA), Universidad CEU San Pablo, Madrid, Spain

**Keywords:** extracellular vesicles, exosomes, extracellular matrix, microenvironment, cell function

## Abstract

Extracellular vesicles (EVs) — including exosomes, microvesicles and apoptotic bodies — have received much scientific attention last decade as mediators of a newly discovered cell-to-cell communication system, acting at short and long distances. EVs carry biologically active molecules, thus providing signals that influence a spectrum of functions in recipient cells during various physiological and pathological processes. Recent findings point to EVs as very attractive immunomodulatory therapeutic agents, vehicles for drug delivery and diagnostic and prognostic biomarkers in liquid biopsies. In addition, EVs interact with and regulate the synthesis of extracellular matrix (ECM) components, which is crucial for organ development and wound healing, as well as bone and cardiovascular calcification. EVs carrying matrix metalloproteinases (MMPs) are involved in ECM remodeling, thus modifying tumor microenvironment and contributing to premetastatic niche formation and angiogenesis. Here we review the role of EVs in control of cell function, with emphasis on their interaction with ECM and microenvironment in health and disease.

## Introduction

Most cell types secrete different types of EVs that can be found in all body fluids, as well as in cell culture supernatant. These vesicles are composed of a lipid bilayer that encloses molecules — lipids, proteins, DNA, mRNA and miRNA — derived from the donor cell. These molecules retain their biological function (Valadi et al., 2007) and may affect the function of recipient cells at a long distance, since they can travel in circulation encapsulated by the lipid bilayer. This concept sheds new light on fundamental process of intercellular communication, beyond the need for direct cell-to-cell contact or secretion of soluble factors that act only on neighboring cells. In addition, this feature puts EVs in focus of biomarker research, since mutation-bearing EVs originating from rare or inaccessible tumor cells can be detected in a liquid biopsy in a non-invasive manner (Garcia-Romero et al., 2017).

There are various types of EVs that can form either at the plasma membrane or at the lumen of intracellular compartment. Large membrane vesicles (1000–5000 nm) are released during the late stages of cell death in the form of apoptotic bodies. Microvesicles, also referred to as microparticles, range between 100 and 1000 nm and arise by outward budding and shedding directly from the plasma membrane. Exosomes, between 30 and 100 nm in diameter, form by inward budding of the endosomal membrane, giving rise to multivesicular bodies, which are subsequently released to extracellular space by fusion of late endosome with the plasma membrane. As a consequence of this mechanism, exosome transmembrane proteins retain the same orientation as that of the donor cell plasma membrane (Chaput et al., 2005), which allows their interaction with recipient cell receptors. These surface proteins include tetraspanins (CD9, CD63, and CD81), integrins, ICAM1 (intercellular adhesion molecule 1) and phosphatidylserine, which is also found on the surface of microvesicles and apoptotic bodies (Théry et al., 2009). Following their recognition by cellular receptors, exosomes can directly fuse with the recipient cell membrane, thus incorporating their membrane proteins to the plasma membrane and delivering their cargo to the cytoplasm of the recipient cell. In addition, exosomes derived from infected macrophages, tumor cells, or antigen-presenting cells, contain antigen-bearing MHC classes I and II molecules, as well as co-stimulatory molecules, that can activate T cells and trigger the immune response (Wolfers et al., 2001; Thery et al., 2002; Giri and Schorey, 2008). EVs thus emerged as important mediators of intercellular communication and became subject of increasing scientific interest in the past decade.

Different EV populations can be separated based on their density, centrifugation speed, or markers expressed on their surface; however, the isolation of pure EV subtypes remains a major challenge (Momen-Heravi et al., 2013; Witwer et al., 2013). In addition, their quantification and characterization mostly rely on markers that can be found in different types of EVs, or may not be expressed by all EVs of the particular type (Théry et al., 2006). It is thus difficult to compare between the results of different laboratories and future studies need to be undertaken to improve and standardize EV isolation and characterization techniques.

The ECM functions as a reservoir of growth factors, which can be released during ECM remodeling and can regulate cell proliferation, migration and organ morphogenesis. Dysregulation of ECM components or aberrant ECM remodeling can lead to various pathologies, including cancer. Recent reports have found growth factors and other soluble mediators, such as TNF-α, EGF, FGF, as well as their receptors, associated with the exosome membrane, suggesting their physiological role in disseminating these soluble factors (Zhang et al., 2006; Sanderson et al., 2008; Seelenmeyer et al., 2008). In addition, EVs carry MMPs with proteolytic activity, which can alter EV content, contribute to ECM degradation and actively participate in tumor progression.

Here, we review the recent advances in our understanding of how EVs mediate cell-to-cell communication and their interaction with ECM components. We also discuss the role of EVs in RNA and protein transfer between cells, influencing the invasion of tumor cells, immune evasion, dissemination of developmental signals during organogenesis and tissue repair, and calcification during bone development and pathological conditions, such arterial plaque and kidney stone formation.

## The Role of EVs in Development and Organogenesis

During development and organogenesis, great coordination needs to be achieved between the cells, ECM and the signaling mechanisms. Developmental signals, including Wnt, Hedgehog, bone morphogenetic proteins, and Notch ligands can be soluble, bound to the ECM or associated to the membrane. Some of these signaling proteins are modified by the addition of a lipid during their biogenesis, hence their solubility and long-range diffusion might be compromised. It has been suggested that EVs could act as vehicles for these signals, allowing cell-to-cell communication and coordinated growth during development. Indeed, recent advances in our understanding of EV biogenesis and function reveal that they are essential mediators of intercellular communication, and thus their role in developmental programming, embryonic induction and organogenesis needs to be highlighted (Valadi et al., 2007; McGough and Vincent, 2016).

The formation of organs, such as salivary glands, teeth, lung, kidney, and mammary glands, is marked by branching morphogenesis processes, in which the interaction between epithelium and mesenchyme needs to be tightly coordinated. The epithelial-mesenchymal interactions occur in both directions, and are primarily regulated by ECM and soluble growth factors, as well as EVs (Puthiyaveetil et al., 2016).

**FIGURE 1:**
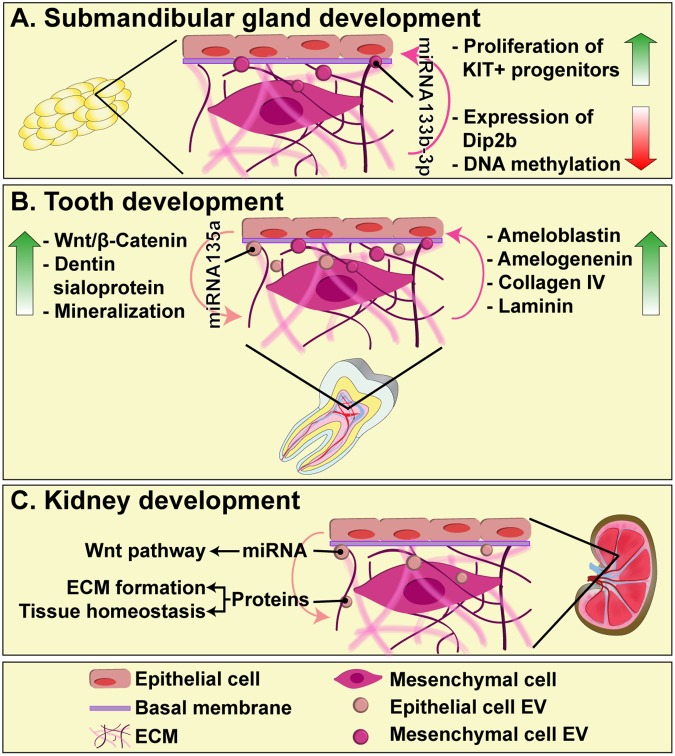
The role of EVs in epithelial-mesenchyme interaction during submandibular gland **(A)**, tooth **(B)**, and kidney **(C)** development. EVs diffuse through the basal membrane and participate in intercellular communication between epithelial and mesenchymal cells, carrying proteins and miRNAs that regulate key events for organogenesis, such as cell proliferation, differentiation and ECM synthesis.

The formation of submandibular glands is a good model to study the communication between mesenchymal and epithelial cells, as well as their interaction with the basal membrane, since it reflects the importance of ECM as a dynamic medium necessary for cell proliferation, apoptosis, differentiation, and migration (Patel et al., 2006; Tucker, 2007). Exosomes derived from mesenchymal cells can pass through the basement membrane and deliver mature forms of miR-133b-3p to epithelial cells, which do not express the primary miRNA. In this manner, miRNA-containing exosomes induce the reduction of Dip2b (Disk-interacting protein 2 homolog B) and DNA methylation in KIT^+^ progenitors, leading to their proliferation (**Figure 1A**) (Hayashi et al., 2017).

During the formation of teeth, exosomes have an indispensable role, since their reciprocal endocytic uptake by epithelial and mesenchymal cells, directly mediates the regulation of cell differentiation and matrix synthesis. Exosomes derived from epithelial cells are able to induce mesenchymal cells to produce dentin sialoprotein and trigger the mineralization processes (Jiang et al., 2017). This occurs through exosomal miR-135a, which leads to activation of Wnt/β-catenin in mesenchymal cells, a critical signaling pathway for matrix synthesis and dentinogenesis (O’Connell et al., 2012). On the other hand, exosomes derived from mesenchymal cells induce epithelial cells to produce collagen type IV and laminin, components of the basement membrane, and scaffolding proteins, such as ameloblastin and amelogenin (Jiang et al., 2017). Therefore, exosomes are essential mediators of the epithelial–mesenchyme interaction that occurs through the basement membrane, and their regulation, including their release and cargo incorporation, are not yet completely understood (**Figure 1B**).

Kidney organogenesis is mediated by the sequential and reciprocal interactions between the epithelial-derived ureteric bud (UB) and metanephric mesenchyme (MM). One of the key players in these interactions is Wnt pathway, by which epithelial UB induces MM transformation and the development of nephrons (Kispert et al., 1998; Pietilä and Vainio, 2014). Very recently, it has been shown that UB-derived exosomes can be taken up by MM cells and transfer their cargo, including miRNAs that play a role in Wnt pathway, as well as proteins involved in ECM organization and tissue homeostasis (Krause et al., 2018). Exosomes thus emerge as an important mechanism of embryonic signaling during kidney development and organogenesis in general, although their content and the mechanism by which they selectively influence different cellular populations, remains to be investigated (**Figure 1C**).

## The Role of EVs in Tissue Repair

Wound repair is a process of re-establishing tissue homeostasis after injury, and it relies on signaling pathways that also act during development. In general, wound repair involves different cell types — including epithelial, immune and endothelial cells — as well as the components of ECM, mostly resulting in scar formation. EVs facilitate coordinating this process, as carriers of pro-resolving mediators. During chronic inflammation, as seen in inflammatory bowel disease, tissue integrity is compromised and epithelial barrier function needs to be re-established. In response to injury, epithelial cells of the intestine secrete EVs that contain a pro-resolving mediator Annexin A1, and thus control wound repair (Leoni et al., 2015).

Cellular therapies, together with the development of biomaterials for the generation of scaffolds, represent the main strategies used in regenerative medicine. Many ongoing studies are directed to elucidate the possible reparative function of EVs and, therefore, evaluate their potential as mediators of cell regeneration.

Diabetes patients commonly present renal disease, characterized by podocyte loss, hypertrophy of mesangial cells (MCs), increase in the ECM protein production and tubulo-interstitial fibrosis (Forbes and Cooper, 2013). Key molecules implicated in this processes are TGF-β and miR-21, which induce collagen and fibronectin production, as well as the up-regulation of matrix protein expression activators, like mTOR (Dey et al., 2012). EVs derived from bone-marrow and human liver stem cells, were shown to transfer miR-222 to MCs and downregulate TGF-β and miR21 in an *in vitro* model of MCs hyperglycaemia, thus serving as potential therapeutic agents to protect MCs from hyperglycaemia-induced damage and collagen production (Gallo et al., 2016).

Osteoporosis treatment is currently limited to approaches stimulating bone formation and anti-resorptive agents (Cheng et al., 2013), thus new studies focused on local transplantation therapies need to be developed. Currently, regenerative strategies for osteoporosis treatment are based on three fundamental lines: mesenchymal stem cells (MSCs), the use of biomaterials for the generation of scaffolds, or the combination of both approaches to achieve a greater regenerative effect (Weinand et al., 2006). However, there are numerous disadvantages when using MSCs as therapy, including the high invasiveness of the procedures needed for harvesting them from donors, possible alteration during cell culture and the presence of MHC proteins that can induce rejection (Izadpanah et al., 2008; Robey, 2011). Advances in the study of cell reprogramming allow the generation of MSCs from induced pluripotent stem cells (iPSCs), which facilitates their management and their use in osteogenesis, although it may also increase the risk of tumorigenesis (Villa-Diaz et al., 2012; Zou et al., 2013). Recently, the osteogenic potential of exosomes derived from hiPSC-MSC (hiPSC-MSC-Exos) has been evaluated in order to overcome the drawbacks related to cell therapy. It was shown that hiPSC-MSC-Exos induce angiogenesis and osteogenesis in ovariectomized rat model, and promote bone regeneration when incorporated on a classical porous β-TCP scaffold (Qi et al., 2016).

Neovascularization is crucial for restoring tissue function after ischemia, although this process is not completely understood. Tissue repair requires the recruitment of proangiogenic mediators and microvesicles, as well as stem and progenitor cells. Many studies focus on endothelial progenitor cell (EPC)-based therapy, since these cells are involved in revascularization processes (Rafii and Lyden, 2003) and may drastically improve regeneration and patients’ outcome (Lara-Hernandez et al., 2010). These cells, nonetheless, require *ex vivo* expansion (Kalka et al., 2000) and may generate HLA incompatibility (Basak et al., 2009). For this reason, the use of EPC-derived EVs emerged as an alternative possibility. During EPC-mediated revascularization, the released EVs induce reprogramming of mature quiescent endothelial cells through horizontal transfer of mRNA, which activates major pathways involved in angiogenesis and leads to endothelial cell proliferation and tissue repair (Deregibus et al., 2007). In addition, EPCs release microvesicles containing angiogenic miRNA-126 and miRNA-296 and thus trigger neoangiogenesis in a murine model of hindlimb ischemia, suggesting the use of EPC-derived microvesicles for treatment of peripheral arterial disease (Ranghino et al., 2012).

Cardiac repair requires endothelial activation, which may be achieved through a proangiogenic factor-inducing therapy. Exosomes contain proteins, such as EMMPRIN (Vrijsen et al., 2010), highlighting the possibility of using EVs as carriers of angiogenesis-stimulating factors for treatment of cardiac ischemia. Indeed, exosomes derived from cardiomyocyte progenitor cells (CMPC) and MSC were shown to carry high levels of EMMPRIN, and may thus regulate VEGF signaling, endothelial cell migration and capillary formation (Vrijsen et al., 2016).

Liver regeneration involves several complex mechanisms, including the mature liver cell reprogramming and proliferation, directed by stem cell populations (Alison et al., 2000; Michalopoulos, 2007). Therefore, obtaining therapies to reduce the recovery time of liver function became a major challenge in this field. In this sense, Dr. Herreras’s group used microvesicles isolated from human liver stem cells (HLSC) as a new approach to improve the degree of regeneration (Herrera et al., 2006). Indeed, in a classical model of 70% hepatectomy in rats, treatment with microvesicles led to increased liver cell proliferation and decreased apoptosis, overall significantly decreasing the liver regeneration time (Herrera et al., 2010).

Extracellular vesicles, as vehicles for proteins and nucleic acids, are thus key mediators of intercellular communication during organogenesis and tissue repair, and their use in regenerative medicine drastically improves current cellular therapies. In addition, the specificity of the uptake by the recipient cells needs to be considered since it increases the potential of EVs as therapeutic vectors.

## The Role of EVs in Bone Calcification

Matrix vesicles (MVs) are particles secreted by a mineralizing tissue to the ECM, and their main function is to promote mineralization. Their *in vitro* and *in vivo* reported size ranges between 0.1 and 2 μm. Furthermore, such MVs may be generated by shedding from plasma membrane or by the endosomal pathway. In this sense, MVs share typical exosomal protein markers, such as the GTPase-Ras family, tetraspanins CD9 and CD63, annexins, integrin receptors and Hsp70 (Shapiro et al., 2015).

Although it is still not clear whether the initial process of mineral formation occurs inside the cell or later in the ECM, several studies point that MVs carry the pre-nucleation complex of calcium phosphate, and that their binding to the ECM attracts other MVs, thus initiating the nucleation phase followed by the apatite formation (Gebauer et al., 2014). Apart from that, MVs also contribute to pathological calcification as found in calcific valvular stenosis, dental plaque, atherosclerosis and calculus renal formation (Anderson et al., 2010).

Bones are formed by collagen fibrils type I, platelets of carbonated hydroxyapatite and calcium phosphate (Mahamid et al., 2011). During osteogenic differentiation most cells die, releasing calcium, MVs, and apoptotic bodies, which become nucleation sites of the hydroxyapatite crystals. Afterwards, the osteoblasts attach to the ECM and differentiate to osteocytes (Grzesiak et al., 2017).

The formation of hydroxyapatite crystals may occur following two main steps. Firstly, when mineral concentrations are imbalanced, calcium and phosphate accumulate and enter the MVs. Secondly, the mineral propagation step occurs when hydroxyapatite crystals are exposed in MV membrane, acting as loci to promote the generation of new crystals (New and Aikawa, 2013). Moreover, it has been reported that the enzyme alkaline phosphatase present in the MVs could help the calcification process (Ali and Griffiths, 1983).

The released MVs could play three different roles in matrix mineralization. The MVs derived by osteoblasts could regulate the ion concentrations, causing the mineralization of the fibrillar collagen ECM (Golub and Boesze-Battaglia, 2007). Another proposed mechanism involves the accumulation of phosphate and calcium within MVs, whose release allow the interaction between MVs and collagen fibrils (Golub, 2009). Finally, the formation of apatite crystals may take place within MVs, which are deposited into collagen fibrils (Harmey et al., 2004).

The degeneration of articular cartilage, also known as osteoarthritis, is characterized by the abnormal calcification in the cartilage matrix. Unfortunately, there is no cure for this disease, and current treatment is only palliative. For that reason, new therapies are being investigated. The injection of MSCs seems like a promising tool, since it shows chondroprotective effect *in vitro* and in mice models. Additionally, it has been suggested that these effects are due to MSC-derived exosomes and microvesicles, which inhibit macrophage activation and chondrocyte apoptosis (Cosenza et al., 2017). Similarly, in a rat model with osteochondral defects, exosome injection promoted cartilage repair, suggesting their possible use as cell-free MSC therapy (Zhang et al., 2016).

Several studies revealed higher amount of MVs in patients compared with healthy controls, suggesting that the presence of these MVs increases extracellular calcium levels and induces hypermineralization (Anderson et al., 2010).

Altogether, we are still far from understanding the precise process of matrix mineralization; therefore, further studies are required to address the function of MVs in those mechanisms.

## The Role of EVs in Cardiovascular Calcification

Cardiovascular diseases are the main cause of death in the world, and vascular calcification is one of the most common complications. In this sense, the lipid accumulation and inflammation of the medium and large arteries precede atherosclerosis, the principal condition leading to heart attacks (Falk, 2006).

The calcification process mediated by the release of MVs appears as an adaptive response to the inflammation process (Anderson et al., 2010). An important variety of MVs, derived from arterial endothelial cells, vascular smooth muscle cells (VSMCs) and macrophages are being associated with the calcification process (Badimon et al., 2017).

In this sense, it is known that in chronic kidney disease macrophage-derived MVs trigger the calcification of atherosclerotic plaques through high concentration of the calcium binding protein S100A9 and annexin V. In addition, after adding Ca^2+^/P to macrophage cell culture, phosphatidylserine translocates to MV external membrane and binds to S100A9-Annexin V complex, promoting hydroxyapatite nucleation. Typical exosomal markers, such as CD9, CD63, and TSG101, are also expressed in this MV population (New et al., 2013).

In these patients, VSMCs increase the secretion of calcifying EVs, decreasing the concentration of extracellular mineralization inhibitors, such as matrix Gla protein or fetuin-A. In addition, elevated levels of TNAP and annexins are found in VSMC-derived EVs, which form microcalcifications when they are delivered into the ECM. This annexin might increase the influx of Ca^2+^ inside MVs and could mediate the interaction of collagen with the ECM (Chen et al., 2008). Afterwards, the calcification propagation is mediated by the collagen fibrils (Bakhshian Nik et al., 2017). Other studies suggest that VSMCs secrete Ca^2+^ and P crystals in the intimal layer of arteries (Kapustin et al., 2017). The mechanism of protein package seems to be highly selective, as cargo of non-calcifying EVs significantly differs from the one seen in calcifying EVs (Shanahan et al., 2012). In addition, under non-calcifying conditions EVs carry miRNAs and inhibitor factors that prevent the calcification pathway (Krohn et al., 2016).

Atherosclerotic plaques display regions with apoptotic cell death, which may be an early event preceding the plaque calcification. In this sense, it has been reported that MVs derived from apoptotic VSMCs contain proapoptotic protein BAX and may initiate the calcification process (Kockx et al., 1998). Furthermore, apoptotic bodies derived from VSMCs undergoing cell death are similar to MVs and could also act as nucleation sites for vascular calcification (Proudfoot et al., 2000).

Other vesicles secreted by devitalized connective tissue cells were also found in calcific valvular stenosis and in the calcification of artificial heart valves (Anderson et al., 2010). Similarly, in the calcific aortic valve disease, valvular interstitial cells secrete pro-calcific EVs that remodel the ECM through the interaction with endothelial cells (Bakhshian Nik et al., 2017).

## The Role of EVs in Renal Calcification

Kidney stones or renal calculi, also referred to as nephrolithiasis, are formed by the nucleation and growth of calcium oxalate (CaOx), calcium phosphate (CaP) or urate crystals (Aggarwal et al., 2013). The incidence of kidney stones is twice as greater in men than in women, and stones submitted by men are more likely to have calcium oxalate crystals, while the stones submitted by women are more likely to have hydroxyapatite (Lieske et al., 2014; Jayachandran et al., 2015). Interestingly, EVs isolated from urine reveal different distribution and protein profile in men and women, which could be related with the above-mentioned gender differences (Jayachandran et al., 2015).

Renal, vascular and bone calcification follow similar pathways, although little is known about the precise mechanism of calcification and matrix synthesis. Similarly to the observations in vascular and bone calcification, renal calcification begins with the release of renal tubular epithelial cell-derived MVs, which serve as nucleation sites in the tubular basement membrane (Anderson et al., 2010). In addition, recent high-resolution microscopy studies have revealed important phenotypic differences between calcifying EVs and MVs in the bone (Bakhshian Nik et al., 2017), reflecting their different cellular origin, biogenesis mechanism and type of mineral they form. Further studies are thus needed to focus on the specific MV subpopulations, as well as the technical methodology to separate and enrich them, for which the different density between calcifying and non-calcifying EVs may play an important role (Hutcheson et al., 2014).

## The Role of EVs in Cancer and Immunity

**FIGURE 2:**
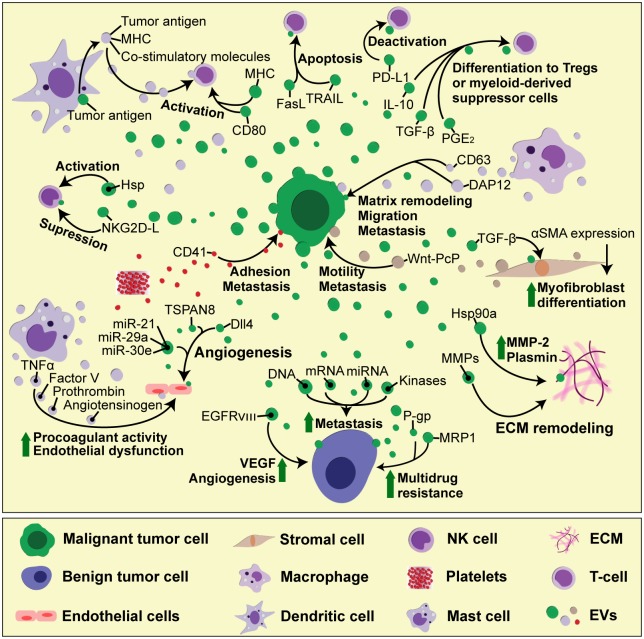
The complexity of cell-to-cell interactions in tumor microenvironment mediated by EVs. During tumor progression, different cell types found in tumor microenvironment — including tumor, stromal, immune and vascular cells — interact reciprocally with each other, as well as with the ECM components, through EVs. These interactions result in immune cell activation or deactivation, which can either hamper or promote tumor growth, depending on the availability of soluble factors, which modulate the microenvironment status. In addition, tumor cell-derived EVs can spread drug resistance and invasive characteristics to other tumor cells, thus boosting tumor growth and the ability to form pre-metastatic niche.

The EVs play an important role in supporting tumor development (**Figure 2**). Most cancer cells release increased amounts of EVs compared to their non-malignant counterparts (Martins et al., 2013). These EVs carry tumor-specific proteins or DNA mutations that can be used as biomarkers in a liquid biopsy (Garcia-Romero et al., 2017). In addition, tumor-derived EVs carry bioactive molecules, such as functional mRNA, which can get transferred to other cells, altering their behavior and contributing to tumor heterogeneity. In this manner, highly malignant cells can change the phenotype of benign tumor cells, increasing their migratory behavior and metastatic capacity (Zomer et al., 2015). In brain tumor, the oncogenic form of epidermal growth factor receptor (EGFRvIII) can be included as EV cargo and transferred between tumor cells, leading to propagation of transforming activity in cells which lack the primary genetic mutation (Al-Nedawi et al., 2008). This non-genetic horizontal transfer mediated by EVs is particularly important for acquiring resistance to chemotherapy. Functional plasma membrane multidrug efflux transporters, such as P-glycoprotein (P-gp) or Multidrug Resistance-Associated Protein 1 (MRP-1), can be shed from resistant cancer cells as cargo of membrane microvesicles and transferred to drug-sensitive recipient cells (Bebawy et al., 2009; Lu et al., 2013). Apart from directly transferring the effector molecules, EVs can also carry intermediary regulators, such as miRNAs or kinases, which then control gene expression and downstream signaling pathways in recipients cells (Gong et al., 2014).

Tumor microenvironment plays a major role in tumor development. It consists of cells, ECM scaffold, tumor-associated vasculature and soluble factors such as growth factors (Joyce and Pollard, 2009). During wound healing and scar formation, TGF-β-activated fibroblasts upregulate the expression of alpha-smooth muscle actin (α-SMA) and differentiate into myofibroblasts, which are responsible for altering tissue architecture. In TGF-β-rich tumor microenvironment, the exaggerated myofibroblast activity leads to fibrosis, ultimately supporting tumor growth, vascularization and metastasis (Tomasek et al., 2002). Prostate cancer-derived exosomes are loaded with functional TGF-β tethered by betaglycan on exosome surface, and can thus induce α-SMA overexpression and persistent fibroblast-to-myofibroblast differentiation (Webber et al., 2010). Highly invasive breast cancer cells secrete exosomes containing molecular chaperone heat shock protein 90a (hsp 90a), which activates extracellular proteases such as MMP-2 and plasmin, thus promoting tumor cell motility and invasion (McCready et al., 2010). Non-tumor cells in tumor microenvironment — such as fibroblasts or activated immune cells — also secrete EVs, which can promote angiogenesis, tumor cell migration and metastasis. EVs derived from tumor-associated fibroblasts cause increased motility and metastatic potential of breast cancer cells (Luga et al., 2012), by stimulating the non-canonical Wnt planar cell polarity (PCP) signaling — a pathway related with rearranging tissue during development (Gray et al., 2011). Platelet-derived exosomes carry integrins, such as CD41 which can increase the adhesion properties in lung and breast cancer cells, thus increasing their metastatic potential (Janowska-Wieczorek et al., 2005). Exosome-mediated transfer of macrophage-derived antigens such as CD163 and DAP12 increases matrix remodeling and migration potential in breast and rectal cancer cells and associates with advanced tumor grade and high rates of metastases (Shabo and Svanvik, 2011). Mast cells are mayor players in IgE-mediated allergic responses, but they also accumulate in tumor microenvironment and secrete MMPs, pro-angiogenic and growth factors, as well as pro- and anti- inflammatory signals, that modify tumor cell proliferation and invasiveness (Stockmann et al., 2014; Maciel et al., 2015). Mast cell-derived exosomes contain TNF-α, angiotensinogen, factor V and prothrombin, which all induce the expression of plasminogen activator inhibitor type 1 (PAI-1) in endothelial cells, causing procoagulant activity and endothelial dysfunction (Al-Nedawi et al., 2005). Acidic pH and hypoxia, hallmarks of tumor microenvironment, have also been reported to modify tumor-derived exosome release and uptake. In acidic conditions, the fluidity and lipid composition of exosomal membrane are changed due to increased sphingomyelin/ganglioside GM3 content, which leads to increased exosome fusion capacity of melanoma exosomes, particularly in metastatic cells (Parolini et al., 2009). Under hypoxia, tumor cells exhibit reduced adhesive properties and increased production of MMPs and invasiveness, accompanied by increased secretion of proteins involved in angiogenesis and immune cell recruitment, all of which found to be enriched in tumor-derived exosomes (Park et al., 2010).

Tumor-derived EVs carry MHC molecules loaded with tumor antigens, as well as co-stimulatory molecules that stimulate antigen-specific T cell responses. In addition, exosomal heat shock proteins (HSP) function as endogenous danger signals that can stimulate NK cell responses (Khalil et al., 2011). Tumor-derived EVs thus have the potential to boost immune responses, providing a promising strategy for anticancer immunotherapy. Indeed, tumor-derived exosomes can serve as antigen source for dendritic cells (DCs), resulting in production of DC-derived exosomes that are able to present antigens and activate T cell-mediated antitumor responses (Pitt et al., 2014; Gu et al., 2015).

However, the cargo of tumor-derived EVs and their immunomodulatory effects seems to depend on tumor microenvironment and the functional status of the immune cells. In most cases, tumors develop different strategies to evade the immune system, and these are reflected in their EV content. Tumor-derived EVs directly participate in immune evasion, for example by generation of suppressive myeloid cells (Valenti et al., 2006), by expressing FasL and inducing T cell death (Andreola et al., 2002; Kim et al., 2005), as well as other suppressive molecules, such as PD-L1, TRAIL, IL-10, and TGF-β, which induce regulatory T cells (Tregs). Tumor-derived exosomes carry NKG2D ligands, which lead to suppression of NK cell function and correlate with poor clinical outcome in patients (Ashiru et al., 2010). The uptake of tumor-derived exosomes blocks DC maturation (Yu et al., 2007), and induces myeloid-derived suppressor cell (MDSC) differentiation through PGE2 (prostaglandin E2) and TGF-β (Xiang et al., 2009).

A number of studies have demonstrated a role for tumor-derived EVs in promoting angiogenesis *in vitro* and *in vivo* (Kim et al., 2002). As mentioned earlier, glioma cells shed EVs containing an oncogenic form of EGF receptor, which increases VEGF expression and contributes to angiogenic signaling in recipient tumor cells (Al-Nedawi et al., 2008). In response to VEGF stimulation, endothelial cells increase motility and proliferation, while simultaneously increasing the expression of Notch ligand Delta-like 4 (Dll4), to inhibit the proliferation of adjacent cells (Phng and Gerhardt, 2009). While this is a well-known mechanism of juxtacrine cell-to-cell inhibition during developmental angiogenesis, VEGF-dependent Dll4 expression in tumor cells promotes tumor growth by enhancing blood vessel diameter and perfusion, which renders these tumors responsive to anti-VEGF therapy with bevacizumab (Li et al., 2007). Interestingly, it has recently been discovered that tumor-derived exosomes contain Dll4 and can thus modulate vessel development in distant recipient cells, providing a new aspect to Notch signaling that does not require direct cell-to-cell contact (Sheldon et al., 2010). The response of endothelial cells to Dll4-containing exosomes seems to differ in a 2D cell culture from a chemically controlled 3D microenvironment with a VEGF concentration gradient (Sharghi-Namini et al., 2014), suggesting that, *in vitro*, just as *in vivo*, tissue microenvironment represents an important factor in the exosome-mediated control of cell function. In another 3D culture model, it was shown that melanoma exosomes can move between endothelial cells by tunneling nanotube networks that contain actin cytoskeleton, similarly as HIV particles or endosomal organelles travel from one cell to another (Rustom et al., 2004; Sowinski et al., 2008; Hood et al., 2009). These exosomes are able to induce tubule branching and the production of endothelial spheroids and sprouts in dose-dependent manner, thus influencing angiogenesis (Hood et al., 2009). Tumor-derived exosomes can also induce endothelial cell activation, proliferation and branching through tetraspanin 8 (formerly known as D6.1A/CO-029), which is associated with poor prognosis in patients with gastrointestinal cancer (Gesierich et al., 2006; Nazarenko et al., 2010). In glioblastoma, one of the most angiogenic solid tumors, several microRNAs (miR-21, miR-29a, and miR-30e) that promote tube formation and angiogenesis are increased within cancer stem cell-derived exosomes, while miR-1, which has a suppressive role, is downregulated (Bronisz et al., 2014; Sun et al., 2017).

Tumor vesicles contain proteinases that are able to degrade ECM and promote tumor invasiveness *in vitro* (Ginestra et al., 1998). Increased tissue factor activity in tumor EVs causes blood coagulation (Zhou et al., 2014), which facilitates tumor cells to adhere to blood vessels and promotes tissue invasion (Dvorak et al., 1981). In addition to endothelial cell proliferation, the tumor induces a distinct blood vessel phenotype characterized by increased permeability that allows tumor cells to enter the circulation and colonize and proliferate at a distant site (Jain, 2005). This vascular leakiness can be induced by melanoma-derived exosomes and represents an early event in pre-metastatic niche formation (Peinado et al., 2012). Bone marrow-derived cells are crucial for this process, and their pro-metastatic phenotype is induced by horizontal transfer of tumor-derived exosomes containing MET oncoprotein (Peinado et al., 2012). Melanoma-derived exosomes are also involved in lymphangiogenesis and lymphatic dissemination, since they carry the metastatic factors that lead to the induction of ECM factors necessary for trapping the metastatic cells in the lymph nodes (Hood et al., 2011). Tumor infiltration and invasion relies mainly on activation of signaling pathways that promote cell migration, ECM remodeling and the expression of MMPs. In addition, this process is accompanied by the production of inflammatory triggers, such as cytokines, chemokines, and reactive oxygen species, which attract bone marrow-derived cells. Alveolar epithelial cells, forming part of the lung stroma microenvironment, express toll-like receptor 3, which recognizes the endogenous small nuclear RNAs carried by tumor-derived exosomes (Liu et al., 2016). As a consequence, alveolar epithelial cells start secreting chemokines, which attracts neutrophils and initiates pro-metastatic inflammatory responses in the lung (Liu et al., 2016).

## Concluding Remarks

Here we reviewed recent research on the role of EVs in intercellular communication and control of cell function, with special emphasis on their interaction with ECM and cell microenvironment. It is becoming evident that EVs can substitute the classical communication through cell–cell contact or protein–receptor interaction, since they carry a greater spectrum of bioactive molecules. In 3D cell culture systems or biological scaffold materials used in regenerative medicine, ECM-bound EVs can influence cell behavior, including cell growth, proliferation, survival, migration and differentiation. These EV properties can be used for tissue repair and regeneration; however, further studies are needed to finely define their cargo signature and functional roles. In addition, current cell therapies impose several disadvantages, such as immunogenicity and tumorigenicity, which may be overcome with the use of EVs for clinical applications. In cancer, therapeutic strategy to inhibit EV secretion might decrease metastatic potential, drug resistance, immune suppression and cancer-associated coagulation disorder. EV-related research is currently flourishing and there is a great interest in deciphering the mechanisms of EV cargo selective packaging and targeting of recipient cells, as well as profiling EV content for biomarker research. However, EV isolation and quantification techniques must be completely standardized before reaching their full potential for clinical applications.

## Author Contributions

AA-S contributed to the design, writing, financial support, and final approval of the manuscript. GR contributed to the design and writing and final approval of the manuscript. NG-R and SE-R contributed to the design and writing of the manuscript. JC-N made the figures. CB-I reviewed the manuscript.

## Conflict of Interest Statement

The authors declare that the research was conducted in the absence of any commercial or financial relationships that could be construed as a potential conflict of interest.

## Abbreviations

ECM: extracellular matrix
EGF: epidermal growth factor
EGFR: epidermal growth factor receptor
EVs: extracellular vesicles
FGF: fibroblast growth factor
MMPs: matrix metalloproteinases
MVs: matrix vesices
PD-L1: programmed death ligand 1
TGF-β: transforming growth factor beta
TNFα: tumor necrosis factor alpha
VEGF: vascular endothelial growth factor
